# Isolation and Evaluation of Rhizosphere Actinomycetes With Potential Application for Biocontrolling *Fusarium* Wilt of Banana Caused by *Fusarium oxysporum* f. sp. *cubense* Tropical Race 4

**DOI:** 10.3389/fmicb.2021.763038

**Published:** 2021-10-25

**Authors:** Lu Zhang, Huixi Zhang, Yating Huang, Jun Peng, Jianghui Xie, Wei Wang

**Affiliations:** ^1^Ministry of Education Key Laboratory for Ecology of Tropical Islands, College of Life Sciences, Hainan Normal University, Haikou, China; ^2^Key Laboratory of Biology and Genetic Resources of Tropical Crops, Ministry of Agriculture, Institute of Tropical Bioscience and Biotechnology, Chinese Academy of Tropical Agricultural Sciences, Haikou, China; ^3^Institute of Environment and Plant Protection, Chinese Academy of Tropical Agricultural Sciences, Haikou, China

**Keywords:** *Streptomyces* isolation, banana *Fusarium* wilt, antifungal activity, plant-growth promotion, biosynthetic gene clusters, GC-MS

## Abstract

Fusarium wilt of banana caused by *Fusarium oxysporum* f. sp. *cubense* tropical race 4 (TR4) is globally one of the most destructive soil-borne fungal diseases. Biological control using environmental microorganisms is considered as an alternative and sustainable strategy. Actinomycetes have the potential to explore biocontrol agents due to their production of diverse metabolites. The isolation and identification of high-efficiency and broad-spectrum antagonistic actinomycetes are the key for the application of biocontrol agents. In the present study, 60 actinomycetes were obtained from the rhizosphere soil of *Machilus pingii* in the primitive ecological natural reserve of Hainan province, China. Seventeen isolates and their extracts exhibited significant antifungal activity against *F. oxysporum* TR4. Particularly, strain BITDG-11 with the strongest inhibition ability had a broad-spectrum antifungal activity. The assay of its physiological and biochemical profiles showed that strain BITDG-11 had the ability to produce IAA and siderophores and had a positive response to gelatin liquefaction and nitrate reduction. Enzyme activities of chitinase, β-1,3-glucanase, lipase, and urease were also detected. Average nucleotide identity calculated by comparison with the standard strain genome of *Streptomyces albospinus* JCM3399 was 86.55% below the novel species threshold, suggesting that the strain could be a novel species. In addition, *Streptomyces* BITDG-11 obviously reduced the disease index of banana plantlets and promoted plant growth at 45 days post inoculation. The higher and lasting expression levels of defense genes and activities of antioxidant enzymes were induced in the roots of banana. Genome sequencing revealed that the *Streptomyces* BITDG-11 chromosome contained large numbers of conserved biosynthesis gene clusters encoding terpenes, non-ribosomal peptides, polyketides, siderophores, and ectoines. Fifteen bioactive secondary metabolites were further identified from *Streptomyces* BITDG-11 extract by gas chromatography–mass spectrometry. Dibutyl phthalate demonstrating a strong antifungal activity was the major compound with the highest peak area. Hence, *Streptomyces* sp. BITDG-11 has a great potential to become an essential constituent of modern agricultural practice as biofertilizers and biocontrol agents.

## Introduction

Banana (*Musa* spp.) is one of the world’s most important staple and cash crops and widely cultivated in 135 countries in tropical and subtropical regions. Annual banana production in the world is estimated at over 145 million tons (Ploetz, 2015a). Due to the lack of effective cross-breeding techniques, commercial banana cultivars are usually multiplied by using vegetative propagation, resulting in a narrow genetic diversity and a susceptible defect to pests and diseases (Wang et al., 2012). *Fusarium* wilt of banana caused by *Fusarium oxysporum* f. sp. *cubense* is one of the most destructive soil-borne fungal diseases and seriously threatens the global banana industry. The causal agents are subdivided into four races based on the banana cultivars infected (Dita et al., 2018). *F. oxysporum* tropical race 4 (TR4) distributed in the main producing areas globally can infect almost all banana cultivars (Pegg et al., 2019). It can be spread by banana material, soil and machinery, irrigation water, etc. (Ploetz, 2015b).

Until now, there is a lack of a commercially effective method of controlling *Foc* TR4 due to the limited knowledge about its disease epidemiology (Bubici et al., 2019; Pegg et al., 2019). Selection of *F. oxysporum* TR4-resistant cultivars seems to be an optimal control strategy, but a productive alternative to the main cultivar Cavendish has not yet been found (Ploetz, 2015b). The poor fertility of Cavendish limits the improvement program through conventional breeding (Zorrilla-Fontanesi et al., 2020). Fungicides lead to chlorotic symptoms and unsatisfactory control efficiency for soil-borne pathogen due to a vascular pathogen penetrating into the plant roots or stems (Nel et al., 2010). Moreover, long-term use of fungicides causes safety concerns and enhances pathogenic resistance (Bubici et al., 2019). Crop rotation is also ineffective as *F. oxysporum* TR4 has a long survival in the soil beyond 20 years, even in the absence of host plants (Ploetz, 2015b). Therefore, before resistant cultivars are obtained, agronomic management approaches will be considered as the main strategy for controlling *Foc* TR4 with *F. oxysporum* TR4.

Recently, biological control using beneficial microorganisms has drawn the attention of scientists because of their economic and environmental advantages (Qi et al., 2019; Jing et al., 2020; Wei et al., 2020). Numerous microbial species have been identified as having the ability for biocontrol, such as *Bacillus* spp., *Burkholderia* spp., *Pseudomonas* spp., *Rhizobium* spp., and *Stenotrophomonas* spp., as well as other genera reported (Bubici et al., 2019). Particularly, *Streptomyces* can develop a symbiotic interaction with plants to promote growth of the host and produce diverse metabolites to inhibit the infection of pathogens (Chen et al., 2018; van der Heul et al., 2018; Bergeijk et al., 2020). *Streptomyces* belonging to the actinomycetes family make up 1–20% of the culturable soil microbes (Olanrewaju and Babalola, 2019). A variety of bioactive compounds are produced with different bioactivities such as antimicrobial, antiviral, and anticancer properties (Bergeijk et al., 2020). Approximately two-thirds of natural antibiotics have been isolated from actinomycetes, and 75% of them are from the *Streptomyces* genus (Franco-Correa et al., 2010). They are the primary antibiotic-producing organisms exploited by the pharmaceutical industry (Olanrewaju and Babalola, 2019). Our previous studies also evidenced that the application of *Streptomyces* sp. could increase the diversity of antagonistic microbes and reduce the disease incidence of *Fusarium* wilt of banana (Jing et al., 2020; Li et al., 2021a; Yun et al., 2021). However, environmental factors limited their stability and biocontrol efficiency against phytopathogens (Saravanan et al., 2003). Thus, the isolation and screening of high efficiency and broad-spectrum antagonistic microorganisms are still key for the application of biocontrol agents.

The extreme niches contribute to the rapid expansion of *Streptomyces* into various species clusters (Bull and Stach, 2007). This ecological diversity drives the evolution of secondary metabolic repertoires and the production of some novel metabolites (Bergeijk et al., 2020). Specialized metabolites were isolated from marine organisms such as arctic *Streptomyces nitrosporeus* (Yang et al., 2013) and desert-dwelling *Streptomyces* sp. (Sayed et al., 2019). In the present study, our aim is to screen and identify actinomycetes with excellently antagonistic *F. oxysporum* TR4 from the primitive ecological environment. We further assessed its efficacy in the management of the pathogen and antifungal mechanisms. Hence, *Streptomyces* have a great potential to become an essential constituent of modern agricultural practice as biofertilizers and biocontrol agents.

## Materials and Methods

### Soil Sampling

The rhizosphere soil of *Machilus pingii* was collected from the primitive ecological nature reserve of ‘Yingge’ mountain (1109°43′05′′E, 19°02′03′′N) in Hainan province, China. The subtropical region has a hot climate in summer (23–36°C) and cold in winter (16–24°C) with an average humidity of 60–80% and annual rainfall of 1,500–2,600 mm. The samples were transported to the laboratory in sterile polythene bags and stored in an insulated container at 4°C.

### Isolation of Actinomycetes

Actinomycetes were isolated by a gradient dilution separation method (Sadeghian et al., 2016). The air-dried soil was ground to powder and passed through a 60-mesh sieve. Five grams of soil samples were dissolved in 45 ml of sterile water. After being incubated at 55°C for 20 min in a water bath, the mixture was cultured at 28°C, 180 rpm, for 1 h. The homogenate was diluted with sterile water into 10^–1^, 10^–2^, and 10^–3^ using a serial dilution method (Li et al., 2021a). One hundred fifty microliters of each dilution were spread on the four-separation agar media, namely, humic acid–vitamin (HV), glucose–aspartic acid (GA), starch casein agar (SCA), and Gause’s No. 1. These media were supplemented with potassium dichromate (50 mg/L) and nystatin (50 mg/L) to inhibit fungal and bacterial contamination. The plates were cultured at 28°C for 5–7 days. The colonies were streaked repeatedly on the ISP2 agar medium for purification until a single colony was obtained. The purified isolates were kept on the Gause’s No. 1 agar medium at 4°C and 20% (v/v) of glycerol at −80°C.

### Preliminary Screening for Antagonistic Actinomycetes Against *F. oxysporum* TR4

The antagonistic activity of actinomycetes against *F. oxysporum* TR4 was initially assayed using an agar diffusion method (Wei et al., 2020). A 5-mm hyphal disk of *F. oxysporum* TR4 was manufactured using a hole puncher and placed in the middle of a potato dextrose agar (PDA, 200 g/L of potato, 20 g/L of dextrose, and 16 g/L of agar) plate. The selected isolate was inoculated in four symmetrical points of about 2.5-cm distance from the pathogen. The *F. oxysporum* TR4 plate without the inoculation of the isolate was used as a control. All plates were cultured at 28°C for 5–7 days under dark conditions. The inhibition activity was measured until the mycelia of *F. oxysporum* TR4 covered the entire surface of the plate. The inhibition percentage was calculated using the following formula:


theinhibitorypercentageofgrowth=[(C--T)/C]×100%

where C and T were growth diameters of tested pathogens in the control and treated plates, respectively.

### Secondary Screening of Antagonistic Actinomycetes Against *F. oxysporum* TR4

Selected actinomycetes with antifungal activity in a preliminary screening were subjected to small-scale fermentation. Briefly, the actinomycete was cultured in a broth medium (15 g of corn flour, 3 g of beef extract, 10 g of yeast extract, 10 g of soluble starch, 10 g of glucose, 0.5 g of K_2_HPO_4_, 0.5 g of NaCl, 2 g of CaCO_3_, 0.5 g of MgSO_4_, pH 7.2–7.4) at 28°C, 180 rpm, for 7 days. The fermentation broth was centrifuged at 13,000 rpm, and the supernatant was mixed with an equal volume of ethanol (95%, v/v). The extract was concentrated using a separating funnel and evaporated with a rotary evaporator (EYELA, N-1300, Japan). The dried extract was diluted with ethanol (50%, v/v) to a final concentration of 10 mg/ml. Antifungal activity was determined by the agar diffusion method as mentioned above after the plates were cultured at 28°C for 7 days.

### Growth Characteristics of the Selected Actinomycetes

To detect the growth characteristics of actinomycetes, the isolate was first cultured in six standard ISP media (yeast extract–malt agar, ISP2; oatmeal agar, ISP3; inorganic salts–starch agar, ISP4; glycerol–asparagine agar, ISP5; peptone yeast–iron agar, ISP6; tyrosine agar, ISP7), PDA, and Gause’s No. 1 (Shirling and Gottlieb, 1968). These plates were incubated at 28°C for 7 days under dark conditions. Colors of substrate and aerial mycelia as well as diffusible pigments were judged by comparing them with the ISCC-NBS color charts (Qi et al., 2019). Some biochemical profiles including nitrate reduction, gelation liquefaction, hydrolysis of cellulose, starch, Tween 20, Tween 40, Tween 80, and urease activities were also measured according to the previous description of Li et al. (2016). IAA production was qualitatively assayed as the development of pink color in the plate. Liquid chrome azurol S (CAS) assay was used to assess siderophore production on ISP2 (Milagres et al., 1999). Chitinase activity was quantitated by measuring the reducing end group of *N*-acetyl glucosamine (Lee et al., 2012). The β-1,3-glucanase activity was measured using laminarin (Sigma, MA, United States) as a substrate (Lee et al., 2012). Resistance evaluation to 20 standard antibiotics was tested by a disk diffusion method (Jing et al., 2020).

### Assay of Broad-Spectrum Antifungal Activity of the Selected Actinomycetes

To detect the broad-spectrum antifungal activity of actinomycete, 18 phytopathogenic fungi were selected, namely, *Fusarium graminearum* Schwabe (ATCC MYA-4620), *Botrytis cinerea* (ATCC 11542), *Cryphonectria parasitica* (ATCC 9414), *Colletotrichum acutatum* (ATCC 56815), *Curvularia fallax* (ATCC 34598), *Colletotrichum fragariae* (ATCC 58689), *Colletotrichum gloeosporioides* (ATCC MYA-456), *Colletotrichum gloeosporioides* (Penz) Saec (ATCC 36351), *Curvularia lunata* (ATCC 42011), *Colletotrichum musae* (ATCC 44422), *Fusarium oxysporum* f. sp. *cubense* race 1 (ATCC 31271), *Fusarium oxysporum* f. sp. *cubense* tropical race 4 (ATCC 76255), *Fusarium oxysporum* (Schl.) f. sp. *cucumerinum* (ATCC 204378), *Fusarium oxysporum* f. sp. *lycopersici* (ATCC MYA-1199), *Colletotrichum gloeosporioides* Penz. (ATCC MYA-4130), *Alternaria tenuissima* (ATCC 96828), *Pyricularia oryzae* (ATCC 52083), and *Colletotrichum fructicola* (ATCC 16103). These phytopathogenic fungi were kept in the National Banana Industry Research and Development Center, China Academy of Tropical Agricultural Sciences, Haikou, China. Antifungal activities of the selected actinomycetes were evaluated based on the radial growth inhibition of phytopathogenic fungi *in vitro* (Wei et al., 2020). The control plates were poured with only phytopathogenic fungi. All experiments were performed in triplicate.

### Stable Characteristics and Safe Evaluation of the Isolate Extract

The extract stock was diluted to 80 μg/ml using sterile water. Seven temperature gradients, namely, 50, 60, 70, 80, 90, 100, and 121°C, were selected to treat the isolate extract for 1 h. Different pH values were prepared from 3 to 10 at an interval of one unit using 1 mol/L HCl or 1 mol/L NaOH. The extract was irradiated by an ultraviolet lamp with an illumination intensity of 100 μW cm^–2^ for 1, 3, 5, 7, 9, 11, and 13 h. Antifungal activity against *F. oxysporum* TR4 was used to evaluate extract stability after treatment of different physical factors. Hemolytic assay of human red blood cells treated with the extract was carried out to test its safety as in our previous description (Li et al., 2021b).

### Genome Sequencing and Molecular Identification of the Selected Actinomycetes

The actinomycete strain with the highest antifungal activity was identified by using a molecular procedure. The isolate was cultured in the ISP2 liquid medium at 28°C for 4 days. Total genomic DNA was extracted using a Rapid Bacterial Genomic DNA Isolation Kit (Biotech Corporation, Beijing, China) according to the standard manufacturer protocol. The complete genome was sequenced using a paired-end sequencing method in the Illumina HiSeq × Ten platform (Illumina, San Diego, CA, United States) by the Shanghai Majorbio Bio-pharm Technology Co., Ltd. A multilocus sequence analysis (MLSA) was performed using 31 housekeeping genes, namely, *dnaG*, *frr*, *infC*, *nusA*, *pgk*, *pyrG*, *rplA*, *rplB*, *rplC*, *rplD*, *rplE*, *rplF*, *rplK*, *rplL*, *rplM*, *rplN*, *rplP*, *rplS*, *rplT*, *rpmA*, *rpoB*, *rpsB*, *rpsC*, *rpsE*, *rpsI*, *rpsJ*, *rpsK*, *rpsM*, *rpsS*, *smpB*, and *tsf*, from the selected actinomycete genome (Brady et al., 2008). The MLSA phylogenetic tree was constructed using the neighbor-joining method in MEGA 7.0 (Kumar et al., 2016). The confidence level was investigated using bootstrap analysis on 1,000 replicates. Average nucleotide identity (ANI) between genomes was calculated using the online OrthoANI (Yoon et al., 2017). The genome sequence of the standard strain (*Streptomyces albospinus* JCM3399) was downloaded from the public genome database of EzBioCloud^1^.

### Genomic Annotation of the Selected Actinomycetes

Sequencing data of the genome were analyzed on an online platform of Majorbio Cloud^2^. Clean sequences were assembled using SOAPdenovo v2.04. The open reading frames (ORFs) and genome annotation were predicted by the Rapid Annotation using Subsystem Technology (Brettin et al., 2015). These genes were annotated using the Clusters of Orthologous Groups (COGs) and Kyoto Encyclopedia of Genes and Genomes (KEGG) (Ogata et al., 1999; Tatusov et al., 2000). Identification of potential biosynthetic gene clusters (BGCs) was proposed by the bioinformatics tool website antiSMASH v4.0.2 software (Weber et al., 2015)^3^. Gene function analysis was performed through manual BLAST on NCBI^4^. The genome sequence data (accession number: SRR15506182) was deposited in the GenBank database.

### Evaluation of Biocontrol and Plant Growth Promotion Under Greenhouse Conditions

The biocontrol and growth-promoting effects of the selected actinomycete were evaluated in greenhouse experiments. The genotype (cv. Brazilian, AAA group) of banana susceptible to *F. oxysporum* TR4 was multiplied by micropropagation. Plantlets with four to five leaves were transferred to soil and grew at 28°C with 70–80% of relative humidity. The selected actinomycete was cultured in a sterilized soybean liquid medium at 180 rpm for 7 days at 28°C. *F. oxysporum* TR4 was inoculated into a PDA broth at 200 rpm for 10 days at 28°C. The conidial suspension was filtered through eight layers of sterile gauze. The spore suspension of *F. oxysporum* TR4 was adjusted to 10^6^ CFU ml^–1^ with sterile distilled water. Roots of banana plantlets were wounded with a scalpel and then dipped into 30 ml of fungal inoculum suspension for 30 min. The fermentation broth of the selected actinomycete (10^6^ CFU g^–1^ soil) was also added to the roots of each plant. Three experimental groups were set, namely, medium treatment (T1), selected actinomycete + *F. oxysporum* TR4 treatment (T2), and *F. oxysporum* TR4 treatment (T3). The banana plantlets were kept in a greenhouse and maintained at approximately 70% of relative humidity at 28°C. The expression levels of defense genes and antioxidant enzyme activity were determined at 1, 2, 3, 4, 5, and 6 days post inoculation (dpi). Plant growth indicators including chlorophyll content, leaf area, leaf thickness, stem diameter, plant height, dry weight, and fresh weight were also measured at 45 dpi (Wei et al., 2020). The disease index was evaluated according to the description of Jing et al. (2020). Data were acquired from three independent experiments, and 36 plantlets were selected for each experiment. All experiments were carried out in triplicates.

### Measurement of Antioxidant Enzyme Activity in Roots of Banana Plantlets

Five grams of frozen root samples were ground in 10 ml of precooled phosphate buffer (50 mM, pH 7.8) containing 2% of polyvinylpyrrolidone. The homogenate was transferred to a 15-ml tube and centrifuged at 15,000 rpm for 15 min at 4°C. The supernatant was used to determine the activity of superoxide dismutase (SOD), catalase (CAT), peroxidase (POD), and polyphenol oxidase (PPO) activities as described by Sun et al. (2013). H_2_O_2_ levels were measured according to the method of Ferguson et al. (1983). The content of malondialdehyde (MDA) was assayed using the thiobarbituric acid reaction method (Zhang et al., 2009).

### Expression Analysis of Defense Genes

Total RNA of banana roots was extracted by the routine TRIzol method. The quality and quantity of RNA were measured by NanoDrop (Thermo Scientific, United States). cDNA was synthesized using the M-MLV reverse transcriptase (Takara, Kyoto, Japan) after treatment with the RNase-free DNase (Takara, Kyoto, Japan). Quantitative real-time polymerase chain reaction (qRT-PCR) was performed in a LightCycler^®^ 480 System (Roche Diagnostics, Mannheim, Germany). A 10-μl reaction system contained 50 ng of first-strand cDNA, 10 μM of forward and reverse primers, and 5 μl of TB Green Advantage qPCR Premix (2×) (Takara, Kyoto, Japan). The reaction program was set as follows: 95°C for 1 min, followed by 40 cycles of denaturation at 92°C for 5 s, annealing at 60°C for 30 s, and extension at 72°C for 30 s. The representative defense marker genes such as *Ma*β*-1,3-Glu* (accession number: AF001523) and mitogen-activated protein kinase 1 (*MaMAPK1*, accession number: XM018826311) were selected. Results were normalized using the expression levels of 18S *rRNA* gene (accession number: U42083). The prime sequences were shown in our previous study (Zhang et al., 2019). The data obtained from different PCR runs were analyzed using the mean of CT values from three biological replicates. The expression ratios were calculated using the 2^–ΔΔCt^ method (Zhang et al., 2019).

### Component Identification of Actinomycete Extract by Gas Chromatography–Mass Spectrometry

The chemical compounds in the extract were identified using gas chromatography–mass spectrometry (GC-MS) as in our previous description (Li et al., 2021b). The strain extract was first dissolved in the chromatographic-grade methanol and filtered through a 0.2-μm filter. The solution was injected into a gas capillary column (DB-FFAP, 30 m × 0.25 mm × 0.25 μm) of a gas chromatography (5973 Inert XL MSD, Agilent, United States). Helium was used as a carrier gas with a flow rate of 1 ml min^–1^. The mass spectrometer (EI with replaceable horn) was operated in the electron ionization (EI) mode at 70 eV with a continuous scan from 50 to 800 *m*/*z*. The peaks were identified by matching the mass spectra with the National Institute of Standards and Technology (NIST, United States) library.

### Statistical Analysis

All experiments were performed in three biological triplicates. Data were expressed as the mean ± standard deviation. The greenhouse experiments were carried out in a randomized design. All data were analyzed using analysis of variance (ANOVA) by the SPSS statistical package (version 22, SPSS Inc., Chicago, IL, United States). The *t*-test compared the mean of an outcome variable for different subjects. The LSD’s multiple range test was applied to determine the significant difference at the level of *p* < 0.05.

## Results

### Actinomycete Isolation

**FIGURE 1:**
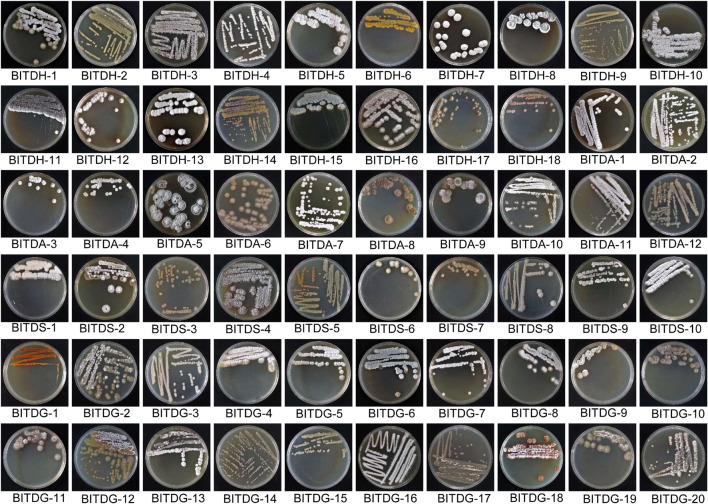
Growth characteristics of 60 actinomycetes isolated from the rhizosphere soil of *Machilus pingii* in the primitive ecological natural reserve.

The primitive nature reserve of ‘Yingge’ mountain possessing great biodiversity was selected for the isolation of antifungal actinomycetes. A total of 60 actinomycetes were isolated from the rhizosphere soil of *M. pingii* using four culture media of HV (18), GA (12), SCA (10), and Gause’s No. 1 (20). Isolated actinomycetes showed well-developed aerial and substrate mycelia in Figure 1. Most aerial mycelia appeared hairy, granular, or powdery. Some isolates produced colored pigments in the substrate and were hard to pick from the media. It was a representative trait of the genus *Streptomyces*. All the isolates were deposited at the National Banana Industry Research and Development Center, the Institute of Tropical Bioscience and Biotechnology, China Academy of Tropical Agricultural Sciences, Haikou, China.

### Antifungal Activity Assay of the Isolated Actinomycetes

Initially, all the isolates were subjected to a primary screening test against *F. oxysporum* TR4. We assessed the inhibition ability of the mycelial growth of *F. oxysporum* TR4. By antagonistic experiments in plates, 17 out of 60 isolates showed significant antifungal activities (Supplementary Figure 1), accounting for 28.3% of the total actinomycetes screened. The inhibitory activity of each isolate was further determined. Eleven actinomycetes owned more than 60% of antifungal activity against *F. oxysporum* TR4. Particularly, the strain numbered BITDG-11 had the best antifungal activity. The inhibition rate reached 80.48 ± 1.49%.

A small-scale fermentation of the 17 isolates with prominent zones of inhibition from the primary screening was carried out. The antagonistic ability of their extracts was further investigated. The results demonstrated that extracts of all the selected isolates showed antifungal activity against *F. oxysporum* TR4, ranging from 27 to 60% (Supplementary Figure 1). The strong inhibition zone sizes were observed in extracts of four isolates (BITDG-11, BITDH-4, BITDG-7, BITDG-19, and BITDA-7). The inhibition rates were 58.44, 45.10, 43.92, 42.89, and 42.62%, respectively. Combined with primary and rescreening results, strain BITDG-11 was selected as an excellent isolate for the subsequent study.

### Growth Characteristics of Strain BITDG-11

The cultural traits of strain BITDG-11 were recorded on eight media selected (Table 1). It had a good growth on ISP2 and a moderate growth on the rest of the media except for ISP4. The color of aerial mycelia varied from white to dark gray. Aerial hypha exhibited dark gray on ISP3, pale yellow on ISP5, and white on ISP2, ISP6, ISP7, PDA, and Gause’s No. 1 media. Light yellow colors of the hyphal substrate were observed on all the media except for light gray on ISP5. Soluble pigment was produced on ISP5, ISP7, and Gause’s No. 1. Production of melanin pigment on ISP7 could be taken as an identification criterion for the genus *Streptomyces* (Atta et al., 2011). Therefore, strain BITDG-11 exhibited typical morphological characteristics of *Streptomyces*.

**TABLE 1:** Growth characteristics of *Streptomyces* BITDG-11 on different media.

Medium	Growth	Aerial mycelium	Substrate mycelium	Soluble pigment	Colony characteristics
Yeast extract malt extract dextrose agar (ISP2)	Good	White	Lemon yellow	No	Hard and wrinkle
Oatmeal agar (ISP3)	Moderate	Dark gray	Rice yellow	No	Powder and dry
Inorganic salts starch agar (ISP4)	Normal	Creamy white	Light yellow	No	Powder and dry
Glycerol asparagine agar (ISP5)	Moderate	Pale yellow	Light gray	Light yellow	Compact and hard
Peptone yeast extract iron agar (ISP6)	Moderate	White	Pale yellow	No	Compact and wrinkle
Tyrosine agar (ISP7)	Moderate	White	Light yellow	Light purple	Powder and loose
Potato dextrose agar (PDA)	Moderate	White	Light yellow	No	Powder and dry
Gause’s No. 1	Moderate	White	Light yellow	Light yellow	Powder and dry

**FIGURE 2:**
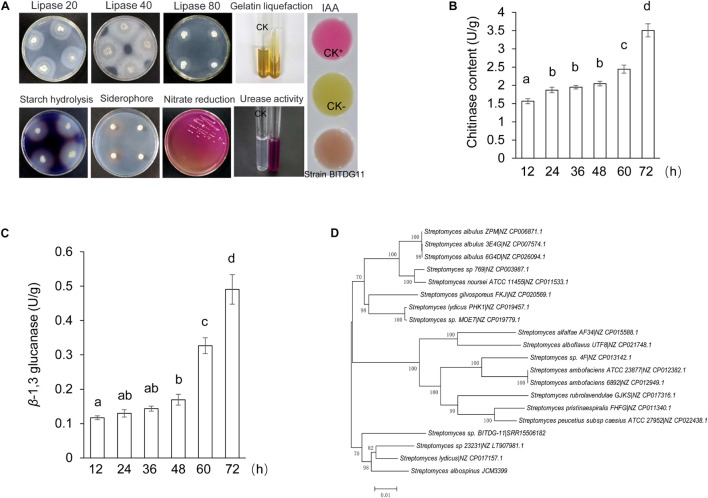
Physicochemical property and identification of *Streptomyces* BITDG-11. **(A)** Physicochemical properties of *Streptomyces* BITDG-11. **(B)** Measurement of chitinase production during the culture process of *Streptomyces* BITDG-11. **(C)** Measurement of β-1,3-glucanase production. Data are the mean values from three biological repeats. Different lowercase letters indicate a significant difference in comparison with the initial time point of the strain growth (LSD’s multiple range test, *p* < 0.05). **(D)** Construction of phylogenetic tree based on MLSA using the neighbor-joining method. The confidence level at all nodes was investigated using bootstrap analysis on 1,000 replicates.

The analysis of physiological and biochemical characteristics showed that strain BITDG-11 had the ability to hydrolyze starch and produce IAA. A positive response was observed to gelatin liquefaction and nitrate reduction. Enzyme activities of lipase and urease were also detected (Figure 2A). The level of chitinase was sharply increased during the exponential phase and reached the peak (3.50 U/mg protein) at 72 h (Figure 2B). The highest level of β-1,3-glucanase (0.49 U/mg protein) was found at 72 h of the incubation period (Figure 2C). Antibiotic susceptibility testing revealed that strain BITDG-11 manifested tolerance to 15 out of 22 kinds of antibiotics and sensitivity to erythromycin, kanamycin, gentamicin, amikacin, neomycin, midenomycin, and ofloxacin (Supplementary Table S1).

### Identification of Strain BITDG-11

Based on the alignment of 16S *rRNA* sequence (1,523 bp, accession number: MW110901), the sequence shared 98.80% of similarity with that of *S. albospinus* NBRC 13846. It confirmed that the strain belonged to a member of the genus *Streptomyces*. However, 58% of the bootstrap value in the neighbor-joining tree indicated that strain BITDG-11 was not distinguished from closely related species using 16S *rRNA* genes.

In order to further identify the species of strain BITDG-11, its whole genome was sequenced. Sequences of 31 housekeeping genes were selected to perform the MLSA. The method was considered as an alternative strategy for defining the *Streptomyces* systematics due to the high efficiency of species resolution and reproducibility (Rong and Huang, 2010). The neighbor-joining tree based on MLSA showed that strain BITDG-11 formed a distinct clade with *S. albospinus* JCM3399 with 70% of bootstrap value (Figure 2D). We further compared the strain BITDG-11 genome (accession number: SRR15506182) with the standard strain genome (*S. albospinus* JCM3399) (Supplementary Table S2). The ANI of 86.55% was below the threshold value of 95–96% for distinguishing a novel species (Richter and Rosselló-Móra, 2009). Thus, strain BITDG-11 might be a novel species of the genus *Streptomyces* and named after *Streptomyces* sp. BITDG-11.

### Assay of a Broad-Spectrum Antifungal Activity

**FIGURE 3:**
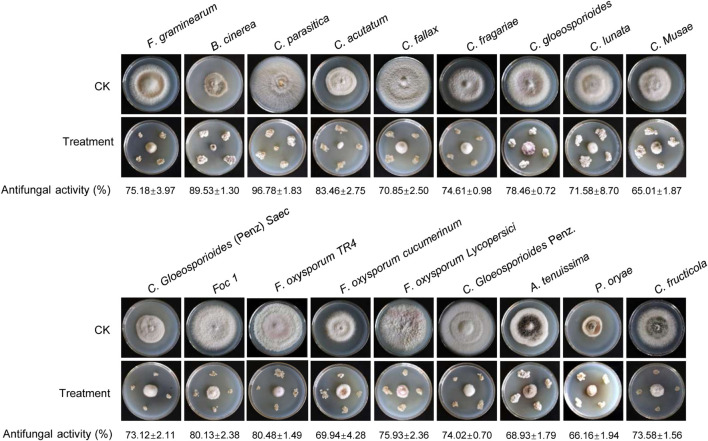
Determination of a broad-spectrum antifungal activity of *Streptomyces* BITDG-11 against the 18 selected phytopathogenic fungi. Different data below the picture indicate antifungal activity of *Streptomyces* BITDG-11 against different phytopathogenic fungi.

To assess whether *Streptomyces* BITDG-11 had a broad-spectrum antifungal activity, 18 fungal pathogens were selected in our study. Compared with the growth diameters of different phytopathogenic fungi in the control plate, the strain had strong antifungal activities against all pathogens selected (Figure 3). The inhibition rates ranged from 65 to 90%. The maximal and minimal inhibition rates were observed against *C. parasitica* (96.78% ± 1.83) and *C. musae* (65.01% ± 1.87), respectively.

### Stability Evaluation of *Streptomyces* BITDG-11 Extract

**FIGURE 4:**
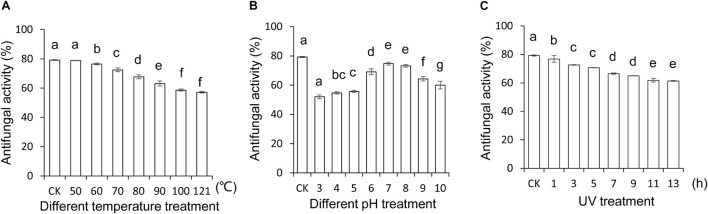
Stability evaluation of the *Streptomyces* BITDG-11 extract after treatment at different temperatures **(A)**, pH values **(B)**, and UV doses **(C)**. Data are the mean values from three biological repeats. Different lowercase letters indicated a significant difference of antifungal activity in comparison with the control (LSD’s multiple range test, *p* < 0.05).

To explore and apply *Streptomyces* BITDG-11 as biopesticides in the future, the stability of the extract expressed as antifungal activity was evaluated after treatment in different temperatures, pH values, and UV doses, respectively. The results showed that different temperatures reduced antifungal activity of the extract against *F. oxysporum* TR4, but 57.05% was kept after treatments at 121°C for 1 h (Figure 4A). For different pH treatments, the strongest antifungal activity was detected at pH 7.0–8.0 and decreased to 52% at pH 3.0 (Figure 4B). Additionally, the extract stability was negatively correlated with the increase of UV dose. A slight decrease was observed in the activity change of the extract treated with UV radiation after 7 h (Figure 4C). Although an obvious decrease (LSD’s multiple range test, *p* < 0.05) of antifungal activity was detected in *Streptomyces* BITDG-11 extract after treatment with different physical factors, more than 50% of residual antifungal activity was retained in all the groups. It suggested that the *Streptomyces* BITDG-11 extract was relatively stable to some extent. To investigate the extract toxicity on eukaryotic cells, hemolytic activity was analyzed. Human red blood cells were measured as the release of hemoglobin after treatment at 37°C for 1 h. Compared with the 100% hemolytic activity of Triton X-100 (0.1%, v/v), no obvious hemolytic activity was observed in the extract treatment group (Supplementary Figure 2).

### *Streptomyces* BITDG-11 Improving Resistance to *F. oxysporum* TR4 and Promoting Growth of Banana Plantlets

**FIGURE 5:**
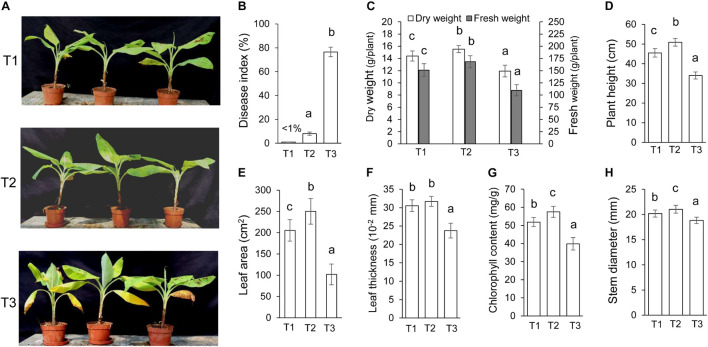
Assay of chlorotic symptoms and plant growth promotion after treatment with *Streptomyces* BITDG-11 at 45 dpi. T1: medium treatment, T2: *Streptomyces* BITDG-11 + *F. oxysporum* TR4 treatment; T3: *F. oxysporum* TR4 treatment. **(A)** Chlorotic symptoms of banana plantlets in different treatment groups. **(B)** Quantitative analysis of disease indexes in different treatment groups. Measurement of physiological indicators including plant dry and fresh weight **(C)**, plant height **(D)**, leaf area **(E)**, leaf thickness **(F)**, chlorophyll content **(G)**, and stem diameter **(H)** in different treatment groups. Error bars indicated standard errors of the means of three repeated experiments. Different letters indicated a significant difference in comparison with the treatment group of *F. oxysporum* TR4 (LSD’s multiple range test, *p* < 0.05).

*Streptomyces* BITDG-11 perfectly inhibited the growth of different phytopathogenic fungi *in vitro*, indicating that it was a promising biocontrol agent. With an objective to evaluate *in vivo* biocontrol efficiency, we analyzed the effects of *Streptomyces* BITDG-11 fermentation broth on the infection inhibition of *F. oxysporum* TR4. By contrast, the bottom leaves of banana plantlets exhibited a significant chlorotic symptom at 45 dpi in the T3 group (Figure 5A). No obvious disease symptom was detected in the group of *Streptomyces* BITDG-11 + *F. oxysporum* TR4. Therefore, the protective treatment with *Streptomyces* BITDG-11 effectively prevented the infection of *F. oxysporum* TR4 and reduced disease index of plants (Figure 5B). We also evaluated actinomycetes and fungal numbers in the rhizosphere of banana seedlings (Supplementary Figure 3). The colony-forming units of *F. oxysporum* TR4 were very low when compared to other treated soils. The results showed that *Streptomyces* BITDG-11 significantly increased competing bacterial and fungal counts along with the pathogen in the soil.

Moreover, various agronomic traits of plant health and condition were measured in Figures 5C–H. *Streptomyces* BITDG-11 significantly (LSD’s multiple range test, *p* < 0.05) increased the fresh and dry weight of banana plantlets at 45 dpi (Figure 5C). Compared to those of control (T1, 45.5 ± 2.2 cm) or *F. oxysporum* TR4 treatment (T3, 34.0 ± 1.8 cm), the height of banana plantlets treated with *Streptomyces* BITDG-11 + *F. oxysporum* TR4 reached 50.9 ± 2.0 cm (Figure 5D). *F. oxysporum* TR4 significantly inhibited the increase of plant agronomic indicators (Figures 5E–H). Similar plant-growth-promoting effects were observed in stem diameters. Although no obvious difference of leaf thickness was detected between dual disposal and controls, a significant increase was found in leaf area and chlorophyll content of plants treated with *Streptomyces* BITDG-11.

### *Streptomyces* BITDG-11 Enhancing the Antioxidant Abilities of Banana Plantlets

**FIGURE 6:**
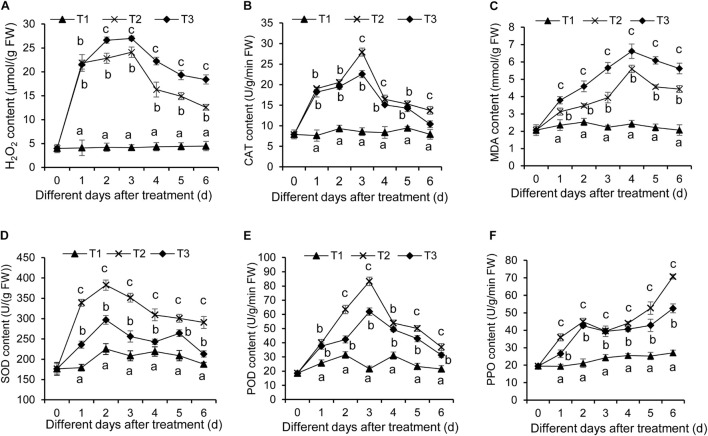
Determination of hydrogen peroxide **(A)**, CAT **(B)**, MDA **(C)**, SOD **(D)**, POD **(E)**, and PPO **(F)** in banana roots after treatment with *Streptomyces* BITDG-11 at different time points. T1–T3 represent different treatment groups as in the description in Figure 5. Error bars indicate standard errors of the means of three repeated experiments. Different letters indicate a significant difference in comparison with the medium treatment group at the same time points (LSD’s multiple range test, *p* < 0.05).

As is well-known, the stress condition could induce the production of reactive oxygen species, thereby resulting in oxidative damage of plant cells. This effect can be assayed by quantifying H_2_O_2_ content and CAT activity. In our study, H_2_O_2_ content in banana roots was increased after *F. oxysporum* TR4 treatment (Figure 6A). Although a similar increase was detected in banana roots treated with *F. oxysporum* TR4 + *Streptomyces* BITDG-11, H_2_O_2_ content was reduced significantly (LSD’s multiple range test, *p* < 0.05) by inducing high CAT activity (Figure 6B). Hence, *Streptomyces* BITDG-11 effectively alleviated the cell membrane damage. It was supported by the fact that MDA content was lower in roots treated with *Streptomyces* BITDG-11 (Figure 6C). Moreover, the strain also increased the higher activities of defense-related enzymes (PPO, POD, and SOD) in comparison with *F. oxysporum* TR4 or control treatment alone (Figures 6D–F). The maximal values of POD, PPO, and SOD were 83.20, 70.67, and 351.31 U/g in the banana roots after inoculation with *Streptomyces* BITDG-11 combined with the challenge with *F. oxysporum* TR4 at 3, 6, and 2 dpi, respectively.

### *Streptomyces* BITDG-11 Increasing the Expression of Defense Genes

**FIGURE 7:**
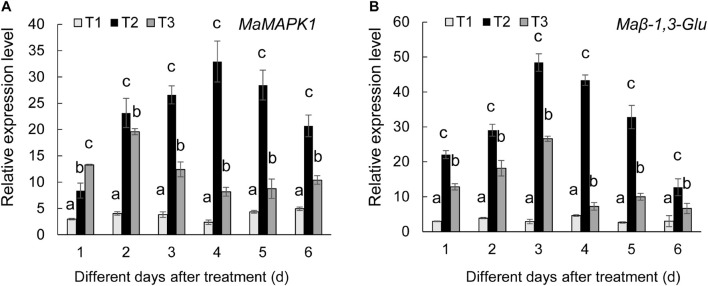
Expression levels of the selected *MaMAPK1* **(A)** and *Ma*β*-1,3-Glu* **(B)** in banana roots after treatment with *Streptomyces* BITDG-11 at different time points. T1–T3 represent different treatment groups as in the description in Figure 5. Error bars indicate standard errors of the means of three repeated experiments. Different letters indicate significant differences in comparison with the medium treatment group at the same time points (LSD’s multiple range test, *p* < 0.05).

To further determine the defense mechanism of *Streptomyces* BITDG-11-induced resistance to *Fusarium* wilt of banana, the transcription levels of *MaMAPK1* (early defense response marker gene) and *Ma*β*-1,3-Glu* (downstream defense marker gene) were analyzed by qRT-PCR (Figure 7). In comparison with control and *F. oxysporum* TR4-only treatment, the transcripts of two tested genes were much stronger in the banana roots treated with *Streptomyces* BITDG-11 + *F. oxysporum* TR4 and reached their maximum values at 4 and 3 dpi, respectively. In addition, high transcription levels of defense genes were kept by *Streptomyces* BITDG-11 treatment until 6 dpi. These results indicated that *Streptomyces* BITDG-11 could prime the banana plants to enhance resistance to *F. oxysporum* TR4 by simultaneously activating the *MAPK*-mediated signaling pathway of defense response.

### Bioinformatics Analysis of the Genome

**FIGURE 8:**
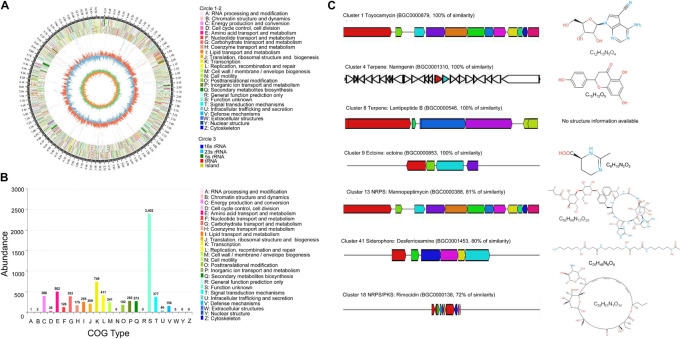
Genome information and function annotation of *Streptomyces* BITDG-11. **(A)** Circular map of *Streptomyces* BITDG-11 genome. From outside to center, ring 1 is the mark of genome size. Rings 2 and 3 represent CDS on the forward/reverse strand. Different colors indicate the functional category of different COGs of CDS. Ring 4 is tRNA and rRNA. Ring 5 shows the G + C content. The outward red part indicates that the GC content of this region is higher than the average GC content of the whole genome. The inward blue part indicates that the GC content of this region is lower than the average GC content of the whole genome, followed by a G + C skew in ring 6. **(B)** COG annotation of *Streptomyces* BITDG-11 genome. **(C)** Biosynthesis gene clusters demonstrating more than 70% similarity with the known sequences.

The *Streptomyces* BITDG-11 genome was sequenced and contained 10,590,458 bp. The genome with 71.78% of GC content included seven *rRNA* genes, 78 *tRNA* genes, and 10,366 coding sequences (CDSs) (Figure 8A). By annotation, 68.45, 28.51, and 57.77% of CDSs were assigned to COG, KEGG, and GO, respectively. For 7,096 genes in COG, the top five categories contained transcription (749), amino acid transport and metabolism (502), energy production and conversion (398), and carbohydrate transport and metabolism (393). Notably, 2,402 of COG genes were clustered into an unknown function category (Figure 8B). The GO annotation showed that these genes were classified mainly into the biological process (2063), cellular component (2113), and molecular function (5023) (Supplementary Figure 4). Interestingly, 78% of genes participated in the regulation of metabolism in KEGG (Supplementary Figure 5). By analysis of the antiSMASH software, 43 BGCs related to biosynthesis of the secondary metabolites were found in genome sequences of *Streptomyces* BITDG-11 (Supplementary Table S3). BGCs included one terpene-type I PKS-nucleoside-NRPS, one type III PKS, one thiopeptide-transatpks-type I PKS-NRPS, five terpene, three NRPS, four type I PKS, one type II PKS, one type I PKS-NRPS, one NRPS-lantipeptide, one lantipeptide-terpene, two lantipeptide, three bacteriocin, one ectoine, two butyrolactone, one terpene-NRPS, one type II PKS-oligosaccharide-type I PKS, one transatpks-NRPS, two siderophore, one indole, etc. Genome analysis further revealed that seven BGCs showed more than 70% of similarity with toyocamycin (100%), naringenin (100%), lantipeptide B (100%), ectoine (100%), mannopeptimycin (81%), desferrioxamine (80%), and rimocidin (72%) (Figure 8C).

### Identification of the Chemical Composition of the *Streptomyces* BITDG-11 Extract

Gas chromatography–mass spectrometry was performed to identify the chemical composition in the *Streptomyces* BITDG-11 extract. In a comparison of their mass spectra with the NIST library, 15 compounds were identified based on their retention time and molecular weight (Table 2). The peak area represented the proportion of the given compound in the total extract. According to the available library data, chemical compounds were identified as tetradecanoic acid, tricosene, *cis*-1-chloro-9-octadecene, pentadecanoic acid, *n*-hexadecanoic acid, linoleic acid ethyl ester, dibutyl phthalate, 1,2-bis(*p*-(trans-styryl)phenyl)-*cis*-ethylene, 3,3′-dihydroxy-4,4′-dinitrohexafluorobiphenyl, 1,2- bis(*p*-(*cis*-styryl)phenyl)-*trans*-ethylene, 4′-(3-(6-methyl-3-pyri dyl)-1-(*p*-tolyl)-2-pyrazolin-5-yl)acetanilide, colchiceinamide, 3,3′-dihydroxy-4,4′-dinitrohexafluorobiphenyl, 2-imidazoline, 3,5-di(4-chlorophenyl)-1-(4-fluorophenyl), naphtho[2,3-c]fura n-1(3H)-one, 4-(3,4-dimethoxyphenyl)-3a,4,9,9a-tetrahydro-6,7-dimethoxy, and bis(2-ethylhexyl) phthalate.

**TABLE 2:** Identification of chemical composition of the *Streptomyces* BITDG-11 extract by GC-MS.

Compounds name	Retention time (min)	Peak area (Ab*s)	Baseline height (Ab)	Absolute height (Ab)	Peak width 50% (min)	Molecular weight (amu)
Tetradecanoic acid	31.323	4,364,641	287,197	437,164	0.369	228.209
Tricosene	33.202	1,720,859	386,105	770,438	0.168	322.36
*cis*-1-Chloro-9-octadecene	33.521	10,411,455	1,506,075	1,927,431	0.235	286.243
Pentadecanoic acid	33.622	12,986,919	1,792,774	2,386,203	0.411	242.225
*n*-Hexadecanoic acid	35.434	54,441,054	8,332,937	9,724,659	0.512	256.24
Linoleic acid ethyl ester	35.97	4,713,086	1,093,523	2,704,558	0.193	308.272
Dibutyl phthalate	37.9	176,228,995	28,511,169	29,838,598	0.277	278.152
1,2-Bis(*p*-(*trans*-styryl)phenyl)-*cis*-ethylene	40.836	556,397	218,880	1,503,957	0.218	384.188
3,3′-Dihydroxy-4,4′-dinitrohexafluorobiphenyl	42.237	242,647	76,237	1,438,498	0.117	383.982
1,2-Bis(*p*-(*cis*-styryl)phenyl)-*trans*-ethylene	42.824	549,444	96,391	1,506,214	0.193	384.188
4′-(3-(6-Methyl-3-pyridyl)-1-(*p*-tolyl)-2-pyrazolin-5-yl) acetanilide	43.067	1,251,949	273,496	1,728,673	0.168	384.195
Colchiceinamide	43.914	365,851	103,654	1,704,206	0.092	384.169
3,3′-Dihydroxy-4,4′-dinitrohexafluorobiphenyl	44.292	47,927	27,767	1,674,508	0.101	383.982
Naphtho[2,3-c]furan-1(3*H*)-one, 4-(3,4-dimethoxyphenyl)-3a,4,9,9a-tetrahydro-6,7-dimethoxy	47.119	1,012,098	226,056	1,885,888	0.151	384.157

*represents the multiplication sign.

## Discussion

*Fusarium* wilt of banana is globally one of the most destructive soil-borne fungal diseases. Fungicides cause serious problems for human health and environment pollution (Bubici et al., 2019). Biological control is considered as an alternative and sustainable strategy. In recent decades, the potential of plant-growth-promoting *Streptomyces* has been revealed for inhibiting pathogen infection and improving crop yield (Dias et al., 2017; Vurukonda et al., 2018). Although several antagonistic *Streptomyces* were investigated as biological control agents (Vurukonda et al., 2018; Jing et al., 2020; Wei et al., 2020; Li et al., 2021a), field application was limited due to the unstable and deficient antimicrobial activity. Therefore, screening highly efficient and broad-spectrum antagonistic *Streptomyces* is still the key for development of biocontrol agents. In the present study, 17 out of 60 actinomycetes obtained from the rhizosphere soil of *M. pingii* had antifungal activity against *F. oxysporum* TR4. Particularly, *Streptomyces* BITDG-11 showed the strongest growth inhibition for *F. oxysporum* TR4 and broad-spectrum antifungal activities against the 18 selected phytopathogenic fungi. In greenhouse experiments, the application of *Streptomyces* BITDG-11 could significantly alleviate the development of leaf chlorotic symptom and decrease the disease incidence of *F. oxysporum* TR4. Similar antagonistic effects of *Streptomyces* were found in controlling other phytopathogenic diseases (Chen et al., 2019; Xu et al., 2019; Sharma and Manhas, 2020; Zhang K. et al., 2020).

The antifungal mechanism of *Streptomyces* BITDG-11 might be related with its ability to produce hydrolytic enzymes including β-1,3-glucanase and chitinase (Figures 2B,C). These enzymes lead to the destruction of the fungal cell wall and leakage of cell contents, inhibiting the growth of phytopathogenic fungi (Hong et al., 2017). Similar reports showed that *Streptomyces cavourensis* SY224 prevented the infection of *Colletotrichum gloeosporioides* by producing hydrolytic enzymes (Lee et al., 2012). Antifungal activity of *Trichoderma harzianum* QTYC77 against *F. oxysporum* f. sp. *cucumerinum* was related to the secretion of chitinase and β-1,3-glucanase (Zhang S. et al., 2020). The cell-free culture filtrate of *Streptomyces violaceusniger* MTCC 3959 exhibited a broad range of antifungal activity against both white rot and brown rot fungi through the production of chitinase and β-1,3-glucanase (Nagpure et al., 2014). Heterologous expression of chitinase from *T. harzianum* increased inhibition activity of *B. cinerea* (Deng et al., 2019). Mycotoxin production enhanced the fungal infection on plants by repressing chitinase gene expression of the biocontrol agent *Trichoderma atroviride* (Lutz et al., 2004). Indeed, *Streptomyces* BITDG-11 had the potential to degrade starch and cellulose on plate experiments. It was supported by the identification of glucanase and amylase genes in the genome of *Streptomyces* BITDG-11. Together, these findings indicated that the hydrolytic enzymes of *Streptomyces* may be linked to their antagonistic activity against phytopathogenic fungi.

Apart from the generated hydrolytic enzymes, *Streptomyces* species were prolific producers of bioactive metabolites (van der Heul et al., 2018; Bergeijk et al., 2020). Several commercial biofungicides were developed such as rhizovit, mycostop, thatch control, and Actinovate^®^ from different *Streptomyces* species (Sharma and Manhas, 2020). Therefore, the antifungal activity of the *Streptomyces* BITDG-11 extract was evaluated against *F. oxysporum* TR4 in the present study. The results showed that the methanol extract could effectively inhibit the mycelial growth of *F. oxysporum* TR4. Although different physical factors were selected to treat the *Streptomyces* BITDG-11 extract, more than 50% of antifungal activity was retained in all the treatment groups, suggesting that the steadily secondary metabolites with antifungal efficiency were produced in the culture filtrate. Similarly, various metabolites were identified from *Streptomyces* as biocontrol agents, and none of them had any negative effect on plants (van der Heul et al., 2018; Olanrewaju and Babalola, 2019; Bergeijk et al., 2020). It was supported by our results that no hemolytic activity was detected in human red cells treated with the *Streptomyces* BITDG-11 extract (Supplementary Figure 2), suggesting that the extract had non-specific cell lytic activity and toxicity to eukaryotic cells. Furthermore, 15 bioactive secondary metabolites were further identified from the *Streptomyces* BITDG-11 extract by GC-MS. Among these compounds, alkanoic acids including tetradecanoic acid, pentadecanoic acid, and linoleic acid were a main type of antifungal production. The previous study reported that tetradecanoic acid had the potential for controlling *Culex quinquefasciatus* and *Aedes aegypti* mosquitoes (Sivakumar et al., 2011). Its isomers can be successfully applied to agriculture and pharmacy industries due to their excellent anti-elastase, anti-urease, and antioxidant activities (Sokmen et al., 2014). The addition of exogenous pentadecanoic acid promoted lipopeptide production and enhanced the antifungal activities of *Bacillus amyloliquefaciens* (Ding et al., 2019). Linoleic acid ethyl ester was critical for fungal response to salicylic acid, mycelial growth, and virulence (Zhang et al., 2017). Particularly, dibutyl phthalate was the major compound with the highest peak number and area in the *Streptomyces* BITDG-11 extract. The compounds produced by *Streptomyces* strain KX852460 had a high antifungal activity against *Rhizoctonia solani* (Ahsan et al., 2017). Other antibacterial and antifungal agents including 9-octadecene, colchiceinamide, and bis-(2-ethylhexyl) phthalate were also detected in microbial culture by the high-performance liquid chromatographic method (Hughes and Davis, 1981; Al-Bari et al., 2005; Thekkangil and Suchithra, 2019). A 9-octadecene compound identified from *Streptomyces albidoflavus* STV1572a contributed to the infection inhibition of *Trichophyton mentagrophytes* (Thekkangil and Suchithra, 2019).

In greenhouse experiments, *Streptomyces* BITDG-11 inhibited the infection of *F. oxysporum* TR4 and promoted the growth of banana seedlings. The possible mechanism might be related to the ability of *Streptomyces* BITDG-11 to produce IAA, siderophore, and antifungal compounds. Siderophore- and IAA-producing actinomycetes in rhizosphere were known to promote plant growth and stress tolerance in main crops (Dias et al., 2017; Vurukonda et al., 2018). Siderophores not only inhibited the growth of phytopathogens but also increased the iron supply to plants and microorganisms (El-Tarabily et al., 2009). This might explain our observation that *Streptomyces* BITDG-11 significantly increased plant height, fresh and dry weight, and chlorophyll content.

Additionally, the interaction of plant and antagonistic agents induces the production of specific root exudates and alters the microbial community diversity (Khamna et al., 2009). We also found that *Streptomyces* BITDG-11 improved the fungal and bacterial abundance to inhibit the growth of *F. oxysporum* TR4 in the rhizosphere soil of banana plantlets. These microbes play an important role in maintaining the stability of the soil ecosystem and reducing plant susceptibility to pathogens (Palaniyandi et al., 2011; Zhou et al., 2019; Trivedi et al., 2020). Accumulated evidences indicated that the plant immune system could be triggered after inoculation with pathogens or beneficial microbes (de Lamo and Takken, 2020). *Streptomyces* BITDG-11 induced higher and lasting expression levels of defense genes in roots of banana seedlings. Moreover, higher activities of antioxidant enzymes such as SOD, CAT, POD, and PPO were detected in the treatment group of *Streptomyces* BITDG-11 + *F. oxysporum* TR4 over pathogen only or control. POD could directly inhibit the spore germination and mycelia growth of some pathogenic fungi like *Pseudocercospora abelmoschi* (Joseph et al., 1998). Enhanced activities of antioxidant enzymes were also reported in bacterium-treated banana plantlets demonstrating tolerance to banana bunchy top disease (Kavino et al., 2007). A recent report showed that combining different *Streptomyces* strains reduced the disease incidence of *Botrytis gray* mold by improving the activity of antioxidant enzymes in chickpea (Vijayabharathi et al., 2018). The higher activity of antioxidant enzymes in banana roots could be another additional mechanism attributing to the increase of the host immune response to pathogens. These findings opened up the possibility of priming defense reactions by *Streptomyces* BITDG-11 inoculation and thus enhanced disease resistance against pathogens.

Genome sequencing further revealed that the *Streptomyces* BITDG-11 chromosome contained a large number of conserved BGC encoding terpenes, non-ribosomal peptides, polyketides, siderophores, and ectoines. These BGCs had been proven to participate in the regulation of antimicrobial activities of *Streptomyces* strains (Bergeijk et al., 2020). Some compounds such as toyocamycin, naringenin, and ectoine analogs showed a 100% similarity with known structures. Toyocamycin produced by *Streptomyces* belonged to a member of the nucleoside antibiotic family. The compound had been recognized as a promising fungicide for the control of plant diseases (Shentu et al., 2018; Ma et al., 2020). Naringenin exhibited both antimicrobial and antioxidant activities (Ng et al., 2019). As a compatible solute and chemical chaperone, ectoine widely synthesized by bacteria enhanced a cellular defense against detrimental effects (Czech et al., 2019).

A detailed survey and analysis revealed that low similarity was found in other BGCs, suggesting *Streptomyces* BITDG-11 had a great potential for producing novel secondary metabolites. It was supported that 33.9% of genes clustered into the unknown function category in COG. The predicted cluster 12 containing 43 necessary genes showed 81% similarity with the biosynthetic gene cluster of mannopeptimycin. The compound was a novel class of lipoglycopeptide antibiotics active against multidrug-resistant pathogens (Magarvey et al., 2006). Cluster 17 exhibited 72% similarity with the biosynthetic gene cluster of rimocidin, a structurally unique type polyketide belonging to a polyene macrolide (Jeon et al., 2016). Rimocidin produced by *Streptomyces rimosus* M527 demonstrated a strong broad-spectrum antifungal activity (Zhao et al., 2019). Cluster 41 had 80% similarity with the known sequence responsible for the biosynthesis of siderophore. The production of siderophores was further confirmed by the appearance of a yellow halo around the colony on CAS agar (Figure 2A). Catechol-siderophore such as coelichelin (cluster 26) identified from the *Streptomyces* species was previously characterized with 2,3-dihydroxybenzoate (2,3-DHB) as a key functional group (Reitz and Butler, 2019). A recent study demonstrated that 2,3-DHB was a common precursor during the biosynthesis of the catecholate siderophores in *Streptomyces* sp. MBT76 (Gubbens et al., 2017). A similar functional crosstalk was also found during the biosynthesis of enterobactin and other secondary metabolites such as benzoxazoles and caboxamycin in other *Streptomyces* species (Cano-Prieto et al., 2015; Losada et al., 2017). The antiSMASH analysis showed a large number of PKS and NRPS gene cluster distributed in the genome of *Streptomyces* BITDG-11, whether a similar crosstalk exists in *Streptomyces* BITDG-11 merits further investigation.

## Conclusion

In our study, 60 actinomycetes were isolated from the rhizosphere soil of *M. pingii*. Among 17 isolates with antagonistic ability against *F. oxysporum* TR4, strain BITDG-11 had high and broad-spectrum antifungal activity. The strain can produce IAA, siderophores, chitinase, β-1,3-glucanase, lipase, and urease. The molecular identification suggested that strain BITDG-11 might belong to a novel species of the genus *Streptomyces*. It obviously promoted the plant growth of banana plantlets and reduced the disease incidence by inducing the expression levels of defense genes and the activities of antioxidant enzymes. Genomic sequencing and analysis revealed that the *Streptomyces* BITDG-11 chromosome contained 43 conserved biosynthesis gene clusters encoding terpenes, non-ribosomal peptides, polyketides, siderophores, ectoines, etc. Among 15 bioactive secondary metabolites identified by GC-MS in the *Streptomyces* BITDG-11 extract, dibutyl phthalate was the major compound with the highest peak area. These results implied that *Streptomyces* BITDG-11 might be developed as a multifunctional biopesticide against a wide range of phytopathogens and as a bioinoculant to enhance plant growth.

## Data Availability Statement

The datasets presented in this study can be found in online repositories. The names of the repository/repositories and accession number(s) can be found in the article/Supplementary Material.

## Author Contributions

LZ, HZ, and WW developed the ideas and designed the experimental plans. WW and JX supervised the research and provided the fund support. LZ, HZ, and YH performed the experiments. JP, WW, and JX provided the materials. LZ, JP and WW analyzed the data. LZ and WW prepared the manuscript. All the authors contributed to the article and approved the submitted version.

## Conflict of Interest

The authors declare that the research was conducted in the absence of any commercial or financial relationships that could be construed as a potential conflict of interest.

## Publisher’s Note

All claims expressed in this article are solely those of the authors and do not necessarily represent those of their affiliated organizations, or those of the publisher, the editors and the reviewers. Any product that may be evaluated in this article, or claim that may be made by its manufacturer, is not guaranteed or endorsed by the publisher.

## Footnotes

1
https://www.ezbiocloud.net/search?tn=Nocardioides

2
www.majorbio.com

3
http://antismash.secondarymetabolites.org/#!/start

4
https://www.ncbi.nlm.nih.gov/

## Supplementary Material

Supplementary Table S1 Antibiotic sensitivity test of *Streptomyces* BITDG-11.

Supplementary Table S2 Calculation of ANI value.

Supplementary Table S3 Genome-wide analysis of *Streptomyces* BITDG-11 for biosynthesis gene clusters of the biosynthesis of secondary metabolites by the online antiSMASH v4.2.0 software.
