# Supplementary material for: Isolation and Evaluation of Rhizosphere Actinomycetes With Potential Application for Biocontrolling Fusarium Wilt of Banana Caused by Fusarium oxysporum f. sp. cubense Tropical Race 4
Source: Front Microbiol. 2021 Oct 25;12:763038. doi: 10.3389/fmicb.2021.763038 (PMC8573349; doi:10.3389/fmicb.2021.763038)
Supplement: Supplementary Table S1 — Antibiotic sensitivity test of Streptomyces BITDG-11. [file Data_Sheet_1.docx]

**Isolation and evaluation of rhizosphere actinomycetes with potential application for biocontrol of Fusarium wilt of banana**

**Lu Zhang, Huixi Zhang, Yating Huang, Jun Peng, Jianghui Xie, Wei Wang**

**^
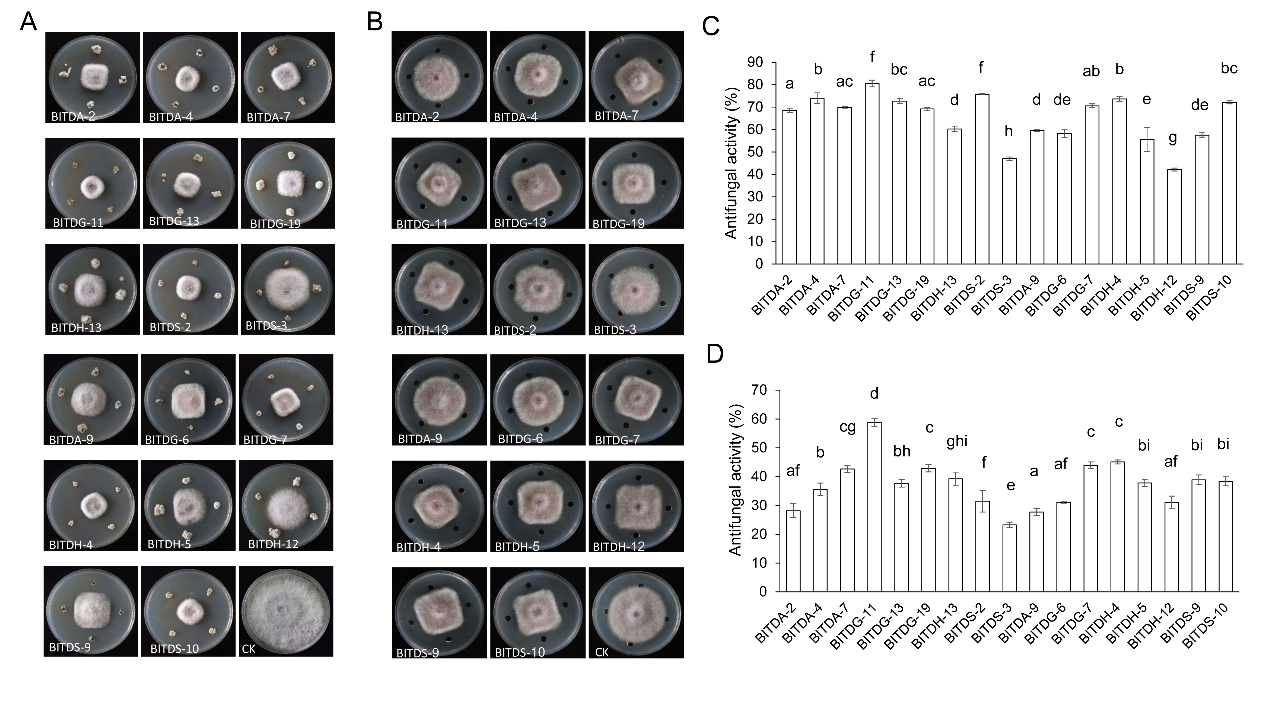
^**

**Figure S1.** Antifungal evaluation of actinomycetes against *F. oxysporum* TR4. A, Growth inhibition of *F. oxysporum* TR4 after antagonist with seventeen isolates. B, Growth inhibition of *F. oxysporum* TR4 after antagonist with extracts of seventeen isolates. C, Quantitative analysis of antifungal activities of different isolates against *F. oxysporum* TR4. D, Quantitative analysis of antifungal activities of different isolate extracts against *F. oxysporum* TR4. Different lowercase letters indicated a significant difference (LSD’s multiple range test, p < 0.05).

**^
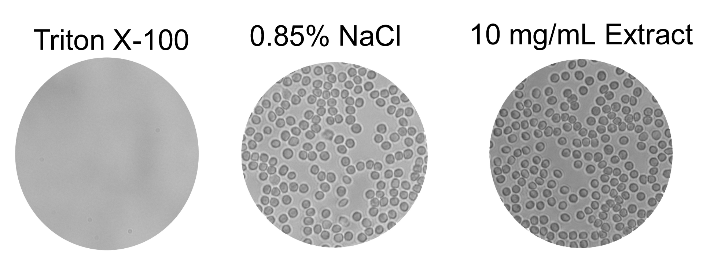
^**

Figure S2 Hemolytic activity assay of extract on human red cells.

**
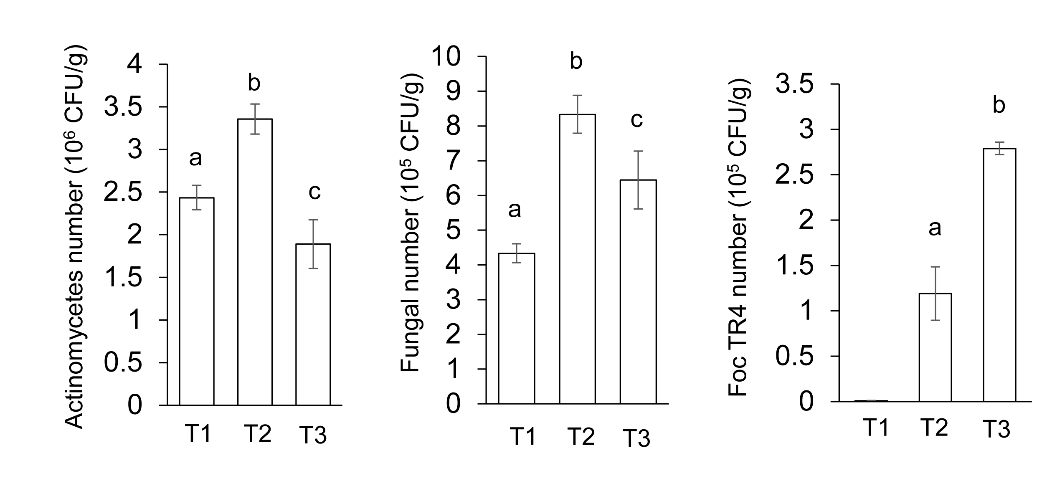
**

Figure S3. Actinomycetes and fungal numbers in the rhizosphere of the different treatment groups. T1: medium treatment, T2: *Streptomyces* BITDG-11 + *F. oxysporum* TR4 treatment; T3: *F. oxysporum* TR4 treatment. Data were the mean values from three biological repeats. Different lowercase letters indicated a significant difference among different treatments according to the LSD’s multiple range test (p < 0.05).

**
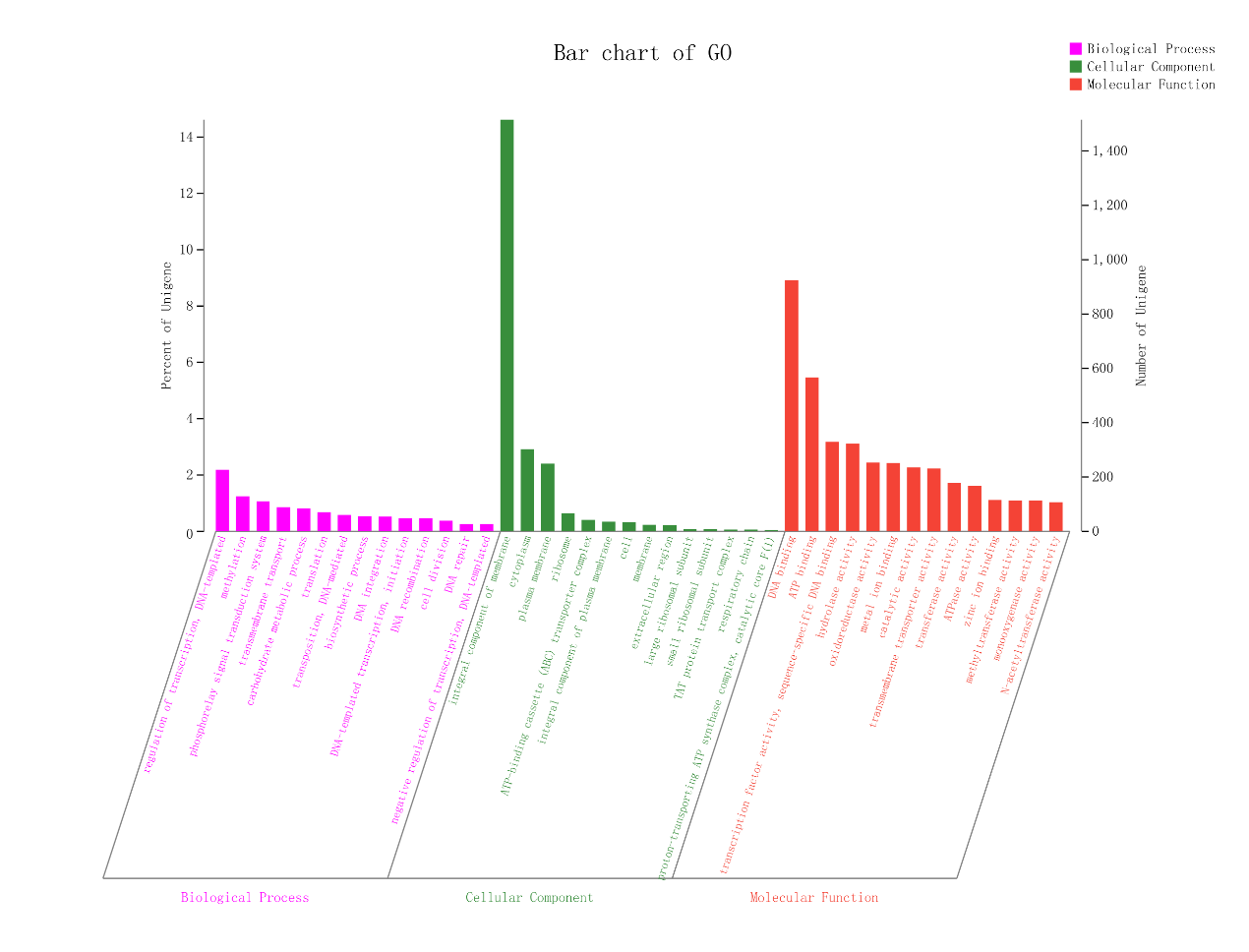
**

Figure S4 GO annotation of *Streptomyces* BITDG-11 genome.

**
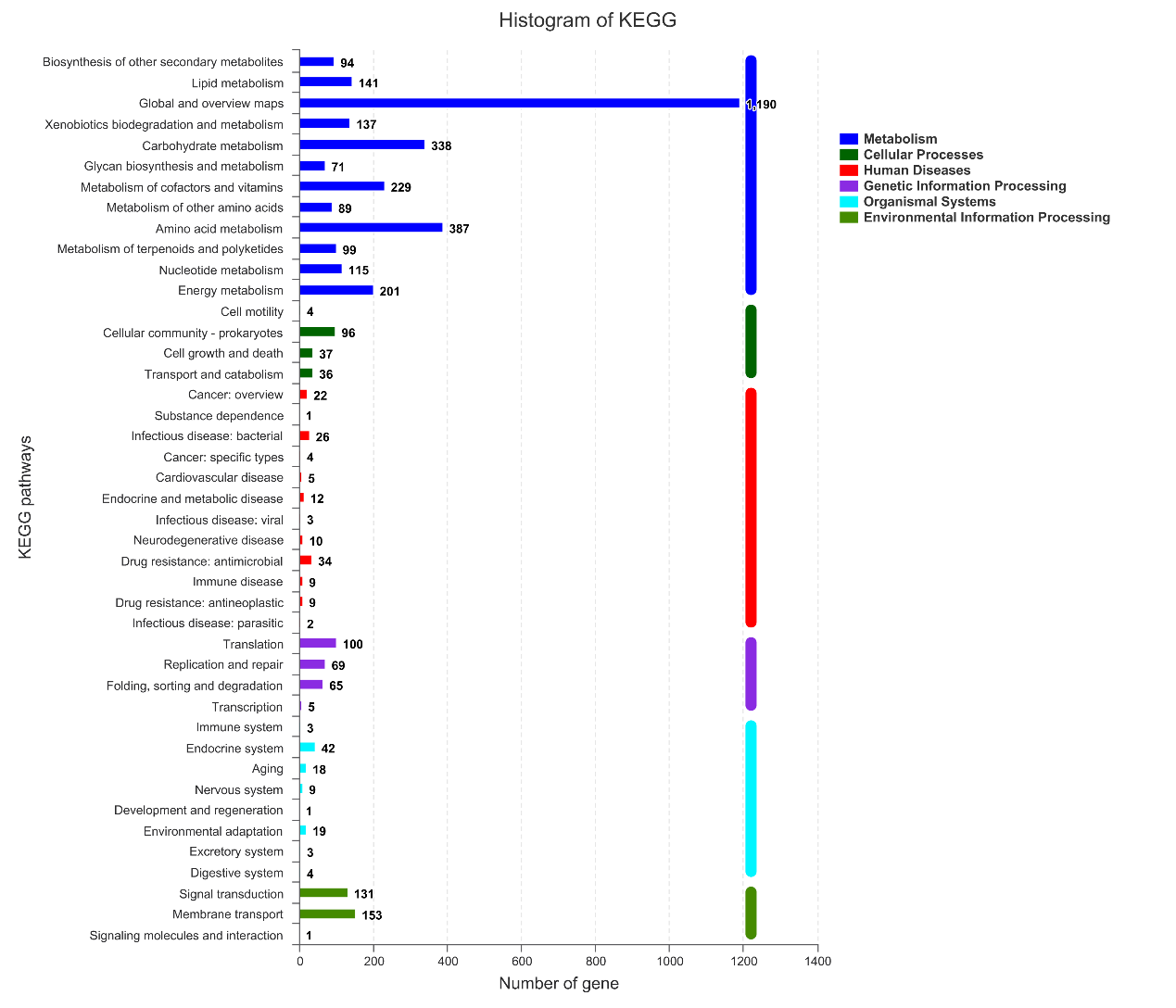
**

Figure S5 KEGG annotation of *Streptomyces* BITDG-11 genome.

Table S1. Antibiotic sensitivity test of strain BITDG-11

| Antibiotic sensitivity (μg disc^-1^) | Results |
| --- | --- |
| Ofloxacin (50) | S |
| Penicillin (100) | R |
| Erythromycin (15) | S |
| Minocycline (30) | R |
| Doxycycline (30) | R |
| Tetracycline (30) | R |
| Neomycin (30) | S |
| Kanamycin (30) | S |
| Gentamicin (10) | S |
| Amikacin (30) | S |
| Cefoperazone (75) | R |
| Ceftriaxone (30) | R |
| Ceftazidime (30) | R |
| Cefuroxime (30) | S |
| Cefradine (30) | R |
| Cefazolin (30) | R |
| Cephalexin (30) | R |
| Midecamycin (30) | R |
| Carbenicillin (100) | R |
| Ampicillin (10) | R |
| Oxacillin (1) | R |
| Piperacillin (100) | R |

Note: S for Sensitivity; R for Resistance.

Table S2 Calculation of ANI value

| Metric | *Streptomyces albospinus* JCM3399 |
| --- | --- |
| ANI value (%) | 86.55 |
| Genome A length (bp) | 10,495,800 |
| Genome B length (bp) | 9,127,980 |
| Average aligned length (bp) | 4,033,447 |
| Genome A coverage (%) | 38.43 |
| Genome B coverage (%) | 44.19 |

**Table S3 Predicted gene clusters of biosynthesis in *Streptomyces* sp. JRGG-11**

| Position | Gene cluster ID | Type | Initial position | Terminal position | Similar biosynthesis gene clusters | Similarity（100%） | Gene number |
| --- | --- | --- | --- | --- | --- | --- | --- |
| Scaffold1 | Cluster1 | terpene-t1pks-nucleoside-nrps | 75108 | 180585 | Toyocamycin_biosynthetic_gene_cluster, other | 100 | 92 |
| Scaffold2 | Cluster2 | bacteriocin | 167268 | 178110 | - | - | 12 |
| Scaffold2 | Cluster3 | butyrolactone | 237753 | 248652 | Griseoviridin_/_viridogrisein_biosynthetic_gene_cluster, nrps | 5 | 11 |
| Scaffold2 | Cluster4 | t3pks | 251719 | 292765 | Naringenin_biosynthetic_gene_cluster, terpene | 100 | 31 |
| Scaffold5 | Cluster5 | thiopeptide-transatpks-t1pks-nrps | 177856 | 290817 | Leinamycin_biosynthetic_gene_cluster, hybrid | 30 | 76 |
| Scaffold6 | Cluster6 | terpene | 55063 | 76268 | Carbapenem_MM_4550_biosynthetic_gene_cluster, other | 6 | 25 |
| Scaffold7 | Cluster7 | nrps | 191025 | 253772 | Enduracidin_biosynthetic_gene_cluster, nrps | 14 | 45 |
| Scaffold8 | Cluster8 | lantipeptide-terpene | 60160 | 92916 | SAL-2242_biosynthetic_gene_cluster, RiPP | 100 | 32 |
| Scaffold9 | Cluster9 | ectoine | 39217 | 49627 | Ectoine_biosynthetic_gene_cluster, other | 100 | 14 |
| Scaffold11 | Cluster10 | nrps | 62092 | 113834 | Telomycin_biosynthetic_gene_cluster, nrps | 8 | 44 |
| Scaffold13 | Cluster11 | t1pks | 1 | 38306 | - | - | 36 |
| Scaffold13 | Cluster12 | nrps | 122200 | 181474 | Mannopeptimycin_biosynthetic_gene_cluster, nrps | 81 | 43 |
| Scaffold14 | Cluster13 | lantipeptide | 15262 | 54582 | Mitomycin_biosynthetic_gene_cluster, other | 3 | 39 |
| Scaffold14 | Cluster14 | bacteriocin | 71812 | 82100 | - | - | 9 |
| Scaffold14 | Cluster15 | t2pks | 134606 | 177107 | Spore_pigment_biosynthetic_gene_cluster, polyketide | 83 | 56 |
| Scaffold16 | Cluster16 | butyrolactone | 126512 | 172297 | Oxazolomycin_biosynthetic_gene_cluster, hybrid | 9 | 42 |
| Scaffold18 | Cluster17 | t1pks | 1 | 28162 | Rimocidin_biosynthetic_gene_cluster, polyketide | 72 | 22 |
| Scaffold18 | Cluster18 | other | 46902 | 87675 | Albonoursin_biosynthetic_gene_cluster, other | 50 | 49 |
| Scaffold19 | Cluster19 | terpene | 36738 | 57388 | SCO-2138_biosynthetic_gene_cluster, RiPP | 14 | 27 |
| Scaffold28 | Cluster20 | siderophore | 61973 | 76653 | - | - | 13 |
| Scaffold32 | Cluster21 | lantipeptide | 43743 | 67043 | - | - | 22 |
| Scaffold35 | Cluster22 | t1pks | 1 | 50540 | Nystatin_biosynthetic_gene_cluster, polyketide | 31 | 15 |
| Scaffold36 | Cluster23 | other | 1 | 24177 | Oxazolomycin_biosynthetic_gene_cluster, hybrid | 15 | 22 |
| Scaffold38 | Cluster24 | terpene-nrps | 7094 | 57677 | Stenothricin_biosynthetic_gene_cluster, nrps | 13 | 57 |
| Scaffold39 | Cluster25 | t2pks-oligosaccharide-t1pks | 1 | 90544 | Saquayamycin_Z_/_galtamycin_B_biosynthetic_gene_cluster, polyketide | 64 | 94 |
| Scaffold40 | Cluster26 | butyrolactone | 72348 | 83283 | Coelimycin_biosynthetic_gene_cluster, polyketide | 8 | 9 |
| Scaffold41 | Cluster27 | terpene | 7874 | 34521 | Hopene_biosynthetic_gene_cluster, terpene | 61 | 28 |
| Scaffold48 | Cluster28 | terpene | 16458 | 38714 | Salinomycin_biosynthetic_gene_cluster, polyketide | 6 | 20 |
| Scaffold49 | Cluster29 | bacteriocin | 67935 | 73652 | - | - | 8 |
| Scaffold51 | Cluster30 | terpene | 49126 | 70056 | Ansatrienin_(mycotrienin)_biosynthetic_gene_cluster, hybrid | 7 | 23 |
| Scaffold53 | Cluster31 | t1pks | 10685 | 57854 | Cylindrospermopsin_biosynthetic_gene_cluster, hybrid | 66 | 49 |
| Scaffold56 | Cluster32 | t1pks-nrps | 1 | 48551 | Cremimycin_biosynthetic_gene_cluster, polyketide | 17 | 50 |
| Scaffold58 | Cluster33 | other | 12644 | 56835 | Meridamycin_biosynthetic_gene_cluster, hybrid | 5 | 49 |
| Scaffold59 | Cluster34 | transatpks-nrps | 1 | 55338 | Oxazolomycin_biosynthetic_gene_cluster, hybrid | 15 | 10 |
| Scaffold61 | Cluster35 | lantipeptide | 22447 | 51080 | Kirromycin_biosynthetic_gene_cluster, hybrid | 3 | 31 |
| Scaffold62 | Cluster36 | lassopeptide | 14432 | 36987 | - | - | 29 |
| Scaffold64 | Cluster37 | t1pks | 16809 | 45426 | Lobosamide_biosynthetic_gene_cluster, t1pks | 15 | 35 |
| Scaffold69 | Cluster38 | nrps | 1 | 42912 | Ansatrienin_(mycotrienin)_biosynthetic_gene_cluster, hybrid | 7 | 33 |
| Scaffold91 | Cluster39 | t1pks | 1 | 20905 | ECO-02301_biosynthetic_gene_cluster, polyketide | 32 | 6 |
| Scaffold93 | Cluster40 | lassopeptide | 3999 | 17792 | - | - | 17 |
| Scaffold95 | Cluster41 | siderophore | 1 | 9246 | Desferrioxamine_B_biosynthetic_gene_cluster, other | 80 | 8 |
| Scaffold99 | Cluster42 | other | 1 | 17005 | - | - | 15 |
| Scaffold135 | Cluster43 | indole | 1 | 8217 | - | - | 11 |
